# Long Range Polymer Chain Dynamics of Highly Flexible Polysiloxane in Solution Probed by Pyrene Excimer Fluorescence

**DOI:** 10.3390/polym10040345

**Published:** 2018-03-21

**Authors:** Janine L. Thoma, Jean Duhamel, Michael J. Bertocchi, Richard G. Weiss

**Affiliations:** 1 Institute for Polymer Research, Waterloo Institute for Nanotechnology, Department of Chemistry, University of Waterloo, Waterloo, ON N2L 3G1, Canada; jlthoma@uwaterloo.ca; 2 Department of Chemistry, Georgetown University, Washington, DC 20057-1227, USA; mjb355@georgetown.edu; 3 Institute for Soft Matter Synthesis and Metrology, Georgetown University, Washington, DC 20057-1227, USA

**Keywords:** long range polymer chain dynamics, pyrene fluorescence, polysiloxane

## Abstract

A poly(dimethylsiloxane-*co*-(3-aminopropyl)methylsiloxane) polymer (PDMS with 20.3 mol % of (3-aminopropyl)methyl siloxane monomer) has been labeled randomly with 1-pyreneacetyl groups to generate a series of polysiloxanes (Py-PDMS) with pyrenyl contents ranging from 0.7 mol % to 5.2 mol % of the total number of structural units. The remainder of the amino groups were acetylated to avoid intra-chain quenching of the excited singlet states of pyrene via exciplex formation with free amino groups while allowing the formation of excimers to proceed. The fluorescence spectra and temporal decays of the Py-PDMS samples were acquired in tetrahydrofuran (THF), *N*,*N*-dimethylformamide (DMF), and dioxane. <*k*^MF^>^blob^, the average rate constant for intra-chain pyrene excimer formation, was determined from the analysis of the fluorescence decays. <*k*^MF^>^blob^ was found to equal 1.16 (±0.13) × 10^9^, 1.14 (±0.12) × 10^9^, and 0.99 (±0.10) × 10^9^ s^−1^ in THF, DMF, and dioxane, respectively, at room temperature. They are the largest values found to date for any polymeric backbone in these solvents. The qualitative relationship found here between <*k*^MF^>^blob^ and the chemical structures of the polymers indicates that the luminescence characteristics of randomly labeled polymers is a very useful method to probe the long range dynamics of chains of almost any polymer that is amenable to substitution by a lumophore.

## 1. Introduction

During the past forty years, monitoring pyrene excimer fluorescence and formation (PEF) has become an important tool to characterize long-range polymer chain dynamics (LRPCD) for polymers dissolved in various media [1,2,3,4,5]. PEF experiments take advantage of the ability of pyrene to form an excimer when an excited pyrene encounters a ground-state pyrene. When the two pyrene labels are covalently attached to a polymer, PEF indicates that the two structural units of the polymer are sufficiently close to enable an encounter. Since PEF experiments are conducted at extremely low polymer concentrations (<10 mg·L^−1^), where inter-chain interactions can be neglected, the rate constant for PEF reflects the ability of one polymer chain to bring two structural units of a same polymer chain, separated by a long contour length, into contact, and thus provides a quantitative measure of LRPCD. Several applications of PEF to characterize LRPCD have been reviewed recently [6,7].

The establishment of reliable techniques capable of probing the magnitude of LRPCD of polymers in solution is important in several aspects of polymer science, including the field of protein folding. The folding of a polypeptide that is initially in a random coil conformation is believed to involve multiple encounters between amino acids that can be located at large separations along a polypeptide sequence. These encounters lead to strong interactions between specific amino acids, resulting in the generation of secondary and then tertiary structural motifs that eventually converge into a structured protein [8]. The entire folding process revolves around the ability of some segments of the polypeptide to diffuse within the spatial frame of the macromolecule and encounter each other. The initial stages of protein folding are thus controlled by LRPCD, and methods capable of characterizing LRPCD quantitatively, such as those based on PEF, are implicitly relevant to the field [7].

To date, benchmarking the ability of PEF to report quantitatively on the LRPCD of pyrene-labeled polymers in solution has been achieved mostly with synthetic polymers due to their wide availability and ease of preparation and handling [7] The PEF experiments conducted on pyrene-labeled linear polymers (PyLLPs) fall into two main categories depending on whether the labels are covalently attached at the ends of a polymer chain having a narrow molecular weight distribution [4,5], or randomly attached along a polymer that can be polydisperse in length [7]. Due to the inherent constraints associated with end-labeled monodisperse polymers, such as extremely low rates of end-to-end encounter for long and rigid chains, the bulk of experiments aiming to characterize LRPCD by PEF have been conducted with linear polymers randomly labeled with pyrene [7]. In one type of PEF experiment, the fluorescence decays of the pyrene monomer and excimer of a series of randomly labeled polymers with different pyrene contents are analysed globally with the Model Free Analysis (MFA), and the LRPCD of the polymer backbone is described by the average rate constant <*k*^MF^>^blob^ that is normalized to account for the different pyrene contents of the PyLLPs [7,9]. So far, the trends obtained with <*k*^MF^>^blob^ on LRPCDs have been found to match closely those expected from the glass transition temperatures (*T*_g_) of polymers in the bulk [7,9]. For instance, polystyrene (PS) and poly(methyl methacrylate) (PMMA), which share similar *T*_g_ values of about 100 °C, also have similar <*k*^MF^>^blob^ values, namely 0.43 and 0.41 ns^−1^ for PS and PMMA, respectively, in tetrahydrofuran (THF) at 22 °C. Poly(methyl acrylate) (PMA), being much less sterically hindered than PMMA, has a much lower *T*_g_ (10 °C) and a <*k*^MF^>^blob^ value (0.88 ns^−1^) that is twice that of PMMA. Similarly, poly(*n-*butyl methacrylate) has a lower *T*_g_ and a higher <*k*^MF^>^blob^ value (27 °C and 0.27 ns^−1^, respectively) than poly(*tert*-butyl methacrylate) (118 °C and 0.19 ns^−1^, respectively) [10]. More recently, the ability of PEF to probe extremely rigid polymeric backbones was applied to study the LRPCD of poly(isobutylene-*alt*-maleic anhydride) (PIMA) [11]. With its rigid succinic anhydride or succinimide ring (depending on the nature of the derivatization) being part of the main chain, PIMA yielded extremely slow LRPCD associated with a very small <*k*^MF^>^blob^ value of 0.047 ns^−1^ in THF at room temperature; that value is one order of magnitude lower than for PS and PMMA.

While PIMA could be viewed as an example of polymeric backbone at the low end of the LRPCD scale, polydimethylsiloxane (PDMS) with a *T*_g_ of −120 °C [10] would certainly be a good backbone example at the higher end of the LRPCD scale. Polysiloxanes are also among the most important industrial polymers due to their wide utility in applications such as high-performance fluids, elastomers, coatings, surface modifiers and separation membranes [12]. To further explore the PEF response on flexible backbones, a series of poly(dimethylsiloxane-*co*-(3-aminopropyl)methylsiloxane), where 20.3 mol % of the structural units were (3-aminopropyl)methyl siloxane, was randomly labeled with 1-pyreneacetic acid (PAA) to yield a series of PyLLPs referred to as Py-PDMS since these polymers have a high dimethylsiloxane content. The steady-state (SSF) and time-resolved (TRF) fluorescence experiments typically conducted on PyLLPs were applied to the Py-PDMS solutions, but their analysis was complicated by the presence of a short oligomeric PDMS species (ODMS). Gel permeation chromatography (GPC) analysis indicated that 11 ± 1 mol % of the pyrene labels were covalently attached onto ODMSs. Since the ODMSs were too short to accommodate more than one pyrene label at the low pyrene-labeling level used in these experiments, all fluorescence spectra showed strong pyrene monomer contributions that prevented the use of the SSF spectra to characterize the LRPCD of the Py-PDMS samples. Fortunately, MFA of the TRF decays acquired with the Py-PDMS samples was able to isolate the contributions of the different pyrene species in solution, so that the fraction of pyrene labels actually attached onto the polymeric PDMS sample could be determined and their behaviour quantified. The <*k*^MF^>^blob^ value retrieved for the Py-PDMS samples in THF equaled 1.16 (±0.13) × 10^9^ s^−1^ which makes Py-PDMS the most flexible polymeric construct ever reported from a MFA of TRF decays acquired with PyLLPs. How this conclusion was reached is described hereafter.

## 2. Materials and Methods

*Materials*: 1-Acetylpyrene (Frinton labs, Hainesport, NJ, USA), morpholine (Alfa Aesar, Haverhill, MA, USA, +99%), sulfur, (Sigma Aldrich, St. Louis, MO, USA, reagent grade, purified by refining), *p*-toluene sulfonic acid, (Acros but distributed by Fisher Scientific: Pittsburgh, PA, USA; monohydrate), 20–25% aminopropylmethylsiloxane—dimethylsiloxane co-polymer (Gelest, Morrisville, PA, USA, AMS-1203; *M*_w_: 20,000), acetic anhydride (Sigma, ≥99%), potassium bicarbonate (Sigma, 99%), 1,1′-carbonyldiimidazole (Fisher, Hampton, NH, USA, 97%, CDI) were used as received. *N*,*N*′-Dicyclohexylcarbodiimide (DCC) and *N*,*N*-dimethylpyridin-4-amine (DMAP) were purchased from Aldrich. Dichloromethane (Fisher, 99%) and dimethylsulfoxide (Fisher, 99%; DMSO) were dried over 4 Å molecular sieves for 3 days before use. Tetrahydrofuran (THF, Caledon, ON, Canada, distilled in glass), *N*,*N*-dimethylformamide (DMF, Sigma, ≥99.8), and dioxane (Sigma, ≥99.5%) were used for the fluorescence measurements.

### 2.1. Synthesis of 1-Pyreneacetic Acid

1-Acetylpyrene was first transformed into 1-pyreneacetothiomorpholide. A mixture of 1-acetylpyrene (2.0 g, 8.2 mmol), sulfur (0.60 g, 19 mmol), morpholine (3 mL, 34 mmol), and *p*-toluene sulfonic acid (0.06 g, 0.35 mmol) were stirred and refluxed at 110–120 °C in a round bottom flask for 8 h. The reflux condenser was outfitted with a tube containing NaOH pellets to neutralize the hydrogen sulfide gas that was produced before it could escape into the environment [13]. The black mixture was cooled, and 20 mL of water wsa added to precipitate the product. The solid was collected by vacuum filtration on a Buchner funnel and washed and triturated with 30 mL of methanol. The orange solid was collected, and dried overnight in vacuo to afford 2.5 g (94% yield), m.p. 192.1–194.5 °C (lit: 192–194 °C) [14].

The morpholide (2.5 g) was added to glacial acetic acid (50 mL) that was heated to reflux. Then, concentrated hydrochloric acid (50 mL) was added slowly and the mixture continued to reflux for 2 h. The reaction was then cooled and additional concentrated hydrochloric acid (20 mL) and water (20 mL) were added to precipitate the product. The crude 1-pyrenylacetic acid was filtered, taken up in 10% K_2_CO_3_ solution (20 mL) and again filtered via a Buchner funnel. Then, the filtrate was acidified with concentrated hydrochloric acid to pH 1 to precipitate the product which was obtained by vacuum filtration to yield 1-pyrenylacetic acid which was recrystallized from chlorobenzene (50 mL) to afford 1.1 g (54%) of yellow-orange crystals, mp 223.2–225.3 °C; (lit: 222.5–225 °C) [14]. Proton and ^13^C NMR spectra of 1-pyreneacetic acid are provided in Figures S1 and S2 in the Supplementary Materials file.

### 2.2. Labeling of Poly(dimethylsiloxane-co-(3-aminopropyl)methylsiloxane) with 1-Pyreneacetic Acid

**Figure 1:**
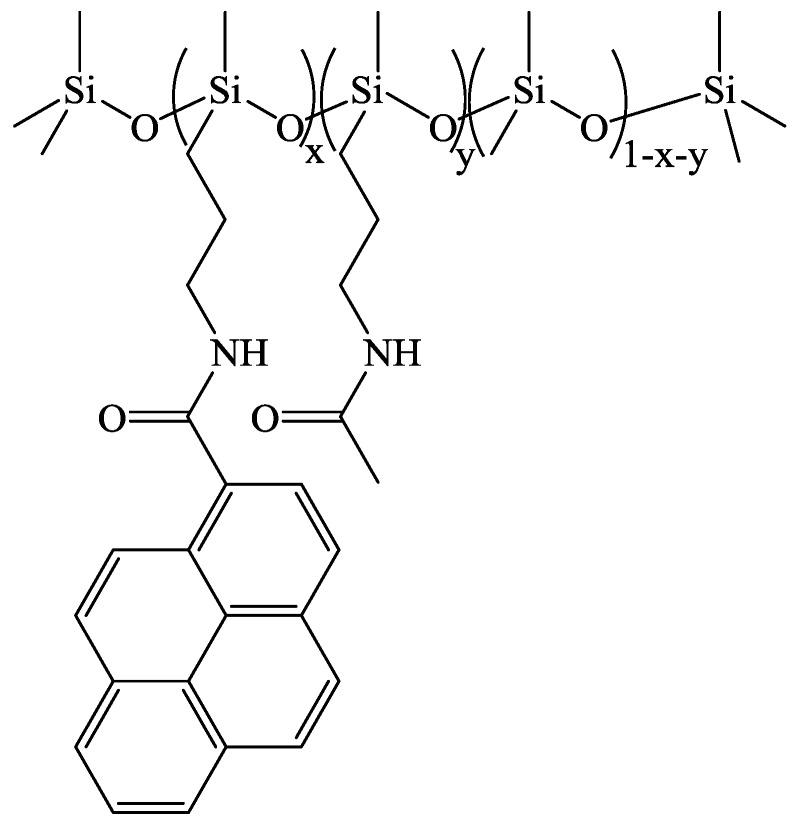
Chemical structure of the pyrene-labeled PDMS, showing the fractions of the monomer unit components. In all cases, *x* + *y* = 0.203.

The synthesis of PDMS labeled with 5 mol % of pyrenyl groups is described below in detail. The syntheses to obtain the other levels of derivatization were done in a completely analogous fashion. A 1:1 molar ratio of 1-pyreneacetic acid (78 mg, 0.30 mmol) and CDI (50 mg, 0.30 mmol) was added to a 50 mL round bottom flask along with 20 mL of dichloromethane (DCM) and 1 mL of DMSO. The contents of the flask were stirred for 2 h at room temperature and the siloxane copolymer (AMS-1203; 500 mg) was added. Stirring continued for 24 h and then potassium bicarbonate (1.0 g; 10 mmol) was added to the flask with the subsequent dropwise addition of acetic anhydride (0.60 mL; 0.60 mmol). After stirring for 2 more h, the reaction mixture was poured into 20 mL water, the layers were separated, and the organic portion was collected. The aqueous phase was washed twice with 20 mL dichloromethane and then the organic phases were combined and reduced with a rotary evaporator set at 40 °C to a residue. A yellow-brown wax was collected and washed twice with methanol to afford 100 mg (19%) of Py-PDMS product. The general chemical structure of the Py-PDMS samples is shown in Figure 1. The ^1^H NMR spectra of the Py-PDMS samples are presented in Figure S3.

### 2.3. Synthesis of the Model Compound, N-Butyl-1-Pyreneacetamide (BPAA, Scheme S1)

Freshly distilled DCM was added to a round bottom flask equipped with a stir bar. 1-Pyreneacetic acid (0.046 g, 0.18 mmol), DCC (0.055 g, 0.26 mmol), and DMAP (0.022 g, 0.18 mmol) were added. *n-*Butylamine (0.017 mL, 0.18 mmol) was then added dropwise to the stirred solution under a nitrogen atmosphere and the mixture was heated overnight at 35 °C. After the reaction mixture had been reduced to a residue on a rotary evaporator, the crude product was passed through a silica gel column using ethyl acetate as the eluent. The fractions containing the product were combined and reduced to a pale yellow solid on a rotary evaporator. The molar extinction coefficient of BPAA in DMF (*ε*_Py_) was found to be 37,300 M^−1^·cm^−1^ at 344 nm, a typical value for a pyrene derivative. The ^1^H NMR, electrospray ionization mass, and fluorescence spectra and decay of BPAA are shown in Figures S4–S6 respectively. The fluorescence spectrum of BPAA exhibited no excimer emission at the 2.5 × 10^−6^ M concentration employed and the fluorescence decay shown in Figure S6B was monoexponential with a long lifetime of 240, 200, and 235 ns in THF, DMF, and dioxane, respectively.

### 2.4. Gel Permeation Chromatography Analyses

**Figure 2:**
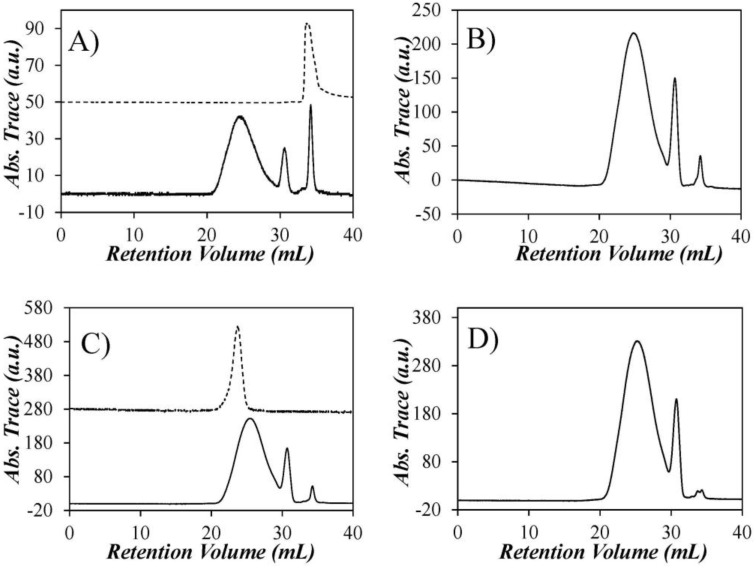
GPC absorbance traces for (**A**) Py(0.7)-PDMS; (**B**) Py(3.4)-PDMS; (**C**) Py(3.5)-PDMS; and (**D**) Py(5.2)-PDMS. The dashed line in Figure 2A represents the GPC trace for PAA while the dashed line in Figure 2C represents the GPC trace of the Py(3.5)-PDMS(f) sample after fractionation of Py(3.5)-PDMS through the GPC column.

All samples were injected into a Viscotek VE 2001 GPC instrument equipped with PolyAnalytik SupeRes mixed bed columns and with a TDA 305 triple detector array (differential refractive index, light scattering, and absorption) using THF as the eluent. A 2600 UV detector module was set at 344 nm and was used to obtain the traces shown in Figure 2. The temperature was set at 35 °C and held constant with a flow rate of 1 mL/min. The presence of pyrene-labeled oligo(dimethyl siloxane) (Py-ODMS) and free 1-pyreneacetic acid (PAA) could be detected by visual inspection. Thus, the Py(3.5)-PDMS sample with 3.5 mol % pyrene label was fractionated using a Waters Millipore M45 preparative GPC instrument equipped with a Jordi gel DVB mixed bed column and a R401 differential refractometer, using THF as the eluent at a flow rate of 3 mL/min. The fraction collected, Py(3.5)-PDMS(f), was then re-injected into the Viscotek GPC 305 to demonstrate that the Py-ODMS and PAA species had been eliminated from the unfractionated Py(3.5)-PDMS sample.

### 2.5. Pyrene Content of Py-PDMS Samples

BPAA was employed to determine the pyrene content of the Py-PDMS samples. A solution of Py-PDMS was prepared with a known mass concentration (*m* in g·L^−1^) in DMF, a solvent which was found to reduce H-bonding between the 1-pyreneacetamide labels of the Py-PDMS samples; see Figure 6D in Results and Discussion. The absorbance of the Py-PDMS solution in DMF at 344 nm (*Abs*(Py)), corresponding to the absorbance of the pyrene labels, was determined using a quartz cuvette with a 1 cm path length. The pyrene content (*λ*_Py_ in mol·g^−1^) was determined from the ratio *Abs*(Py)/[*ε*_Py_ × *m*] where *ε*_Py_ is the molar extinction coefficient of BPAA. In turn, *λ*_Py_ could be expressed according to Equation (1), which accounted for all pyrene species expected to be present in the Py-PDMS solution.

(1) $$\lambda_{Py}=\frac{\left[\mathrm{Py}-\mathrm{PDMS}]+[\mathrm{Py}-\mathrm{ODMS}]+[\mathrm{PAA}\right]}{([\mathrm{Py}-\mathrm{PDMS}]+[\mathrm{Py}-\mathrm{ODMS}])M_{\mathrm{SiN}\mathrm{Py}}+[\mathrm{SiN}]M_{\mathrm{SiN}}+[{\mathrm{Me}}_{2}\mathrm{Si}]M_{\mathrm{Si}}+[\mathrm{PAA}]M_{\mathrm{PAA}}}$$

In Equation (1), ([Py-PDMS] + [Py-ODMS]), [SiN], [Me_2_Si], and [PAA] are the molar concentrations of the (3-aminopropyl)methyl siloxane capped with 1-pyreneacetic acid of molar mass *M*_SiNPy_ (359.48 g·mol^−1^), the acetyl capped (3-aminopropyl)methyl siloxane of molar mass *M*_SiN_ (159.26 g·mol^−1^), the dimethylsiloxane monomers of molar mass *M*_Si_ (74.15 g·mol^−1^), and 1-pyreneacetic acid of molar mass *M*_PAA_ (260 g·mol^−1^), respectively. Taking advantage of the fact that the PDMS sample contained 20.3 mol % of (3-aminopropyl)methylsiloxane units (as determined by titration of the amine groups with aqueous HCl [15]), and assuming that the pyrene contents of Py-PDMS and Py-ODMS are the sam, and that the GPC analysis yielded the molar fraction of PAA present in each Py-PDMS sample, the molar fraction *x* of structural units that were labeled with PAA could be determined precisely. A complete derivation for the determination of *x* can be found in the Supplementary Materials. Accordingly, the pyrene contents of the four Py-PDMS samples were 0.7, 3.4, 3.5, and 5.2 mol %.

### 2.6. Mass Spectrometry

The mass spectrum of BPAA was acquired using a ThermoFisher Scientific Q-Exactive mass spectrometer (Bremen, Germany). Positive ion Electrospray (ESI) was performed with a ThermoFisher Scientific Q-Exactive hybrid quadrupole-orbitrap mass spectrometer. Accurate mass determinations were performed at a mass resolution of 70,000 with lock mass correction. Samples were infused at 10 μL/min in 1:1 methanol: water + 0.1% formic acid.

### 2.7. UV-Vis Absorption

The absorption spectra used to determine the pyrene content of the Py-PDMS samples and the Py-PDMS concentration in the solutions prepared for fluorescence measurements were acquired with a Varian Cary 100 Bio spectrophotometer (Victoria, Australia).

### 2.8. Py-PDMS Solution Preparation for Fluorescence Measurements

All Py-PDMS solutions were prepared with a concentration of pyrene labels equal to 2.5 × 10^−6^ M, a concentration sufficiently low to ensure the absence of inter-chain interactions. Nitrogen was bubbled gently through the Py-PDMS solutions for 30 min to remove dissolved molecular oxygen before sealing the cell containers with a Teflon stopcock.

### 2.9. Steady-State Fluorescence

All fluorescence spectra were acquired on a Photon Technology International steady-state fluorometer. The instrument was equipped with an Ushio UXL-75 Xenon lamp and a PTI 814 photomultiplier detection system. The fluorescence intensities of the deoxygenated Py-PDMS solutions were monitored from 350 to 600 nm at a right-angle geometry and with an excitation wavelength of 344 nm. The fluorescence intensities between 372 and 378 nm and between 500 and 530 nm were integrated to obtain relative fluorescence intensities of the pyrene monomer (*I*_M_) and excimer (*I*_E_), respectively, that were ratioed to yield the expression, (*I*_E_/*I*_M_)^SSF^, which reflects the efficiency of a given Py-PDMS sample at forming excimers.

### 2.10. Time-Resolved Fluorescence

All fluorescence decays of the deoxygenated Py-PDMS solutions were obtained with a 5000U IBH time-resolved fluorometer. The solutions were excited at 344 nm with a 340 nano LED and the fluorescence decays of the monomer and excimer emissions were acquired by setting the emission monochromator at 375 and 510 nm and using cut-off filters at 370 and 495 nm, respectively. The cut-off filters were placed on the emission side of the fluorometer to prevent light scattered by the polymer solutions from reaching the detector. The instrument response function was obtained from the signal scattered off a Ludox solution and by setting the detection wavelength equal to 344 nm. A repetition rate of 500 kHz and a time-per-channel of 2.04 ns/ch were employed to acquire the fluorescence decays over 1024 channels with 20,000 counts at the decay maximum.

### 2.11. Fluorescence Decay Analysis

The monomer and excimer fluorescence decays were fitted with, respectively, Equations (2) and (3) according to the model free analysis (MFA). The standard MFA of fluorescence decays acquired with polymers randomly labeled with pyrene assumes the existence of four pyrene species in solution. These pyrene species are those that form excimer by diffusive encounter with a ground-state pyrene (*Py*_diff_*), that do not form excimer because they are located in sections of the polymer with low pyrene concentrations (i.e., they emit as if they were free in solution and with the natural lifetime *τ*_M_ of the pyrenyl label (*Py*_free_*)), and that are pre-associated with a ground-state pyrene to form a well-stacked (*E*0*) or poorly stacked (EL*) pyrene dimer that emits with a shorter (*τ_E_*_0_) or longer (*τ*_EL_) excimer lifetime, respectively. Equation (2) describes the diffusive encounters of the pyrene labels as a sum of exponentials and leads to the expression of the excimer given in Equation (3). The pre-exponential factors *a*_i_ are normalized to unity in Equation (2). More detailed explanations about the derivation of the MFA can be found in earlier reviews [6,7].

(2) $${\left[Py_{}^{*}\right]}_{\left(t\right)}=({\left[Py_{\mathrm{diff}E0}^{*}\right]}_{\left(t=0\right)}+{\left[Py_{\mathrm{diff}D}^{*}\right]}_{\left(t=0\right)})\times \sum_{i=1}^{n}a_{i}\times \exp(-t/\tau_{i})+{\left[Py_{\mathrm{free}}^{*}\right]}_{\left(t=0\right)}\times \exp(-t/\tau_{M})$$

(3) $$\begin{array}{c} {\left[E*\right]}_{\left(t\right)}=-{\left[Py_{\mathrm{diff}E0}^{*}\right]}_{\left(t=0\right)}\times \sum_{i=1}^{n}a_{i}\frac{\frac{1}{\tau_{i}}-\frac{1}{\tau_{M}}}{\frac{1}{\tau_{i}}-\frac{1}{\tau_{E0}}}\;\exp(-t/\tau_{i})\\ +\left({\left[E0*\right]}_{\left(t=0\right)}+{\left[Py_{\mathrm{diff}E0}^{*}\right]}_{\left(t=0\right)}\times \sum_{i=1}^{n}a_{i}\frac{\frac{1}{\tau_{i}}-\frac{1}{\tau_{M}}}{\frac{1}{\tau_{i}}-\frac{1}{\tau_{E0}}}\right)\times \exp(-t/\tau_{E0})\\ -{\left[Py_{\mathrm{diffEL}}^{*}\right]}_{\left(t=0\right)}\times \sum_{i=1}^{n}a_{i}\frac{\frac{1}{\tau_{i}}-\frac{1}{\tau_{M}}}{\frac{1}{\tau_{i}}-\frac{1}{\tau_{\mathrm{EL}}}}\;\exp(-t/\tau_{i})\\ +\left({\left[EL*\right]}_{\left(t=0\right)}+{\left[Py_{\mathrm{diffEL}}^{*}\right]}_{\left(t=0\right)}\times \sum_{i=1}^{n}a_{i}\frac{\frac{1}{\tau_{i}}-\frac{1}{\tau_{M}}}{\frac{1}{\tau_{i}}-\frac{1}{\tau_{\mathrm{EL}}}}\right)\times \exp(-t/\tau_{\mathrm{EL}}) \end{array}$$

The monomer and excimer decays were fitted globally with Equations (2) and (3). The parameters were optimized with the Marquardt-Levenberg algorithm [16]. The fits were considered acceptable when *χ*^2^ values were <1.3 and residuals and autocorrelation of the residuals were randomly distributed around zero. The parameters retrieved from the MFA of the decays are listed in Tables S1–S3 of the Supplementary Materials file.

The parameters retrieved from the MFA of the fluorescence decays with Equations (2) and (3) could be used to find the molar fractions of all pyrene species in solution. This derivation, which was complicated by the presence of pyrene-labeled ODMS and free 1-pyreneacetic acid in the solution, has been described in Supplementary Materials file.

Also, the pre-exponential factors *a*_i_ and decay times *τ*_i_ in Equation (2) could be employed to calculate the average rate constant <*k*> for pyrene excimer formation as shown in Equation (4). Within the MFA framework, <*k*> is a pseudo-unimolecular rate constant that depends on the local pyrene concentration. As such, it increases with the pyrene content of a PyLLP. The normalization of <*k*>, as shown in Equation (5), yields <*k*^MF^>^blob^, which has been shown to be independent of pyrene content and to reflect accurately the LRPCD for a broad range of polymer backbones.

(4) $$<k>=\frac{1}{\tau_{M}}-\frac{\sum_{i=1}^{n}a_{i}}{\sum_{i=1}^{n}a_{i}\tau_{i}}$$

(5) $$<k^{MF}{>}^{blob}=\frac{1-f_{\mathrm{Mfree}}}{x}\times <k>$$

The parameters retrieved from the MFA of the fluorescence decays could also be combined to yield an absolute measure of the ratio of the fluorescence intensity of the excimer to that of the monomer, (*I*_E_/*I*_M_)^TRF^ (Equation (6)).

(6) $${\left(I_{E}/I_{M}\right)}^{\mathrm{TRF}}=\frac{(f_{\mathrm{diff}E0}\tau_{E0}+f_{\mathrm{diffEL}}\tau_{\mathrm{EL}})<k><\tau >+f_{E0}\tau_{E0}+f_{\mathrm{EL}}\tau_{\mathrm{EL}}}{(f_{\mathrm{diff}E0}+f_{\mathrm{diffEL}})<\tau >+f_{\mathrm{free}}\tau_{M}}$$

## 3. Results and Discussion

The GPC traces of each of the Py-PDMS samples were recorded at an excitation wavelength of 344 nm (Figure 2). The broad peak centered at 25 mL in all GPC traces is for the Py-PDMS sample, while the spike at approximately 30 mL is thought to be the Py-ODMS species, followed by a spike at approximately 34 mL corresponding to residual PAA. The assignment of the spike eluting last was confirmed by injecting a known sample of PAA into the GPC (Figure 2A). The molar fractions of pyrene labels present as Py-PDMS, Py-ODMS, or PAA in the samples were determined by integration of the peak areas in the GPC traces (Table 1). They indicate that 11 ± 1 mol % of the pyrene labels attached to the PDMS belong to the Py-ODMS species. As shown in the upper portion of Figure 2C, it was possible to collect a fraction that was free of Py-ODMS and PAA (Py(3.5)-PDMS(f)) by collecting the front part of the peak of the Py(3.5)-PDMS sample. The Py(3.5)-PDMS(f) sample was employed later to validate the corrections to account for the presence of Py-ODMS and PAA that were applied to the fluorescence data with the unfractionated Py-PDMS samples.

**Figure 3:**
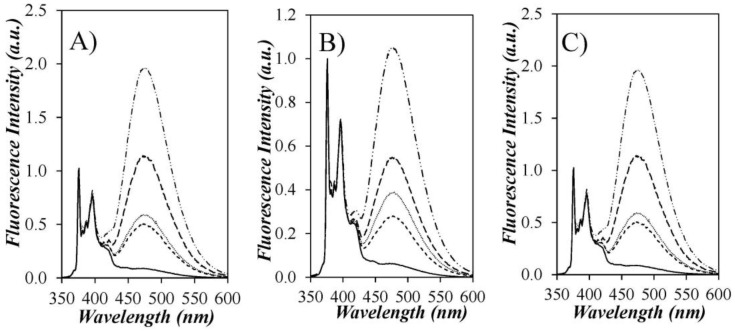
Fluorescence spectra of (—) Py(0.7)-PDMS, (- - -) Py(3.4)-PDMS, (_ .. _ .. _) fractionated Py(3.5)-PDMS(f), (⋅⋅⋅⋅⋅⋅) Py(3.5)-PDMS, and (_ _ _) Py(5.2)-PDMS normalized at the 0-0 transition at 375 nm in (**A**) THF; (**B**) DMF; and (**C**) dioxane. λ_ex_ = 344 nm, [Py] = 2.5 × 10^−6^ M.

Since ODMS is too short to accommodate more than one pyrene label per chain, these oligomers do not form excimers and act as a fluorescent impurity in the characterization of the LRPCD of the Py-PDMS samples. Their negative impact on the fluorescence measurements is clearly illustrated in Figure 3, where the fluorescence spectra of all Py-PDMS samples are presented, including that of Py(3.5)-PDMS(f), the fractionated Py(3.5)-PDMS sample. The fluorescence spectra were normalized at 375 nm, the wavelength for the 0-0 emission band of the pyrene monomer. The normalized fluorescence spectra of the non-fractionated Py-PDMS samples showed a continuous increase in PEF with increasing pyrene content. However, since the SSF spectra cannot distinguish among the different pyrene species in solution, the extent of PEF probed in a SSF spectrum is affected by the emission from the Py-ODMS and PAA species.

How the presence of Py-ODMS affected the fluorescence spectra is clearly illustrated in the fluorescence spectrum obtained for Py(3.5)-PDMS(f). As shown in Figure 2C, the Py(3.5)-PDMS(f) sample was obtained by collecting the solution eluting between 21 and 25 mL following an injection of Py(3.5)-PDMS into the preparative GPC. In so doing, the contributions from the Py-ODMS and PAA species that were present in the Py(3.5)-PDMS sample and eluted in the GPC trace at 30 and 34 mL, respectively, were eliminated in Py(3.5)-PDMS(f). Since these two pyrene species contributed mostly to the fluorescence of the pyrene monomer, the excimer signal in the normalized SSF spectrum of Py(3.5)-PDMS(f) was 3.3, 2.7, and 2.9 times larger than that for the unfractionated Py(3.5)-PDMS sample in THF, DMF, and dioxane, respectively. Such a major difference in the measured fluorescence intensity of the excimer leads to the conclusion that the fluorescence spectra obtained from the unfractionated Py-PDMS samples do not reflect accurately the PEF from pyrene labels covalently attached to PDMS. Consequently, our study focused mainly on the analysis of the TRF decays.

It is also noteworthy that the Py-PDMS samples yield the least PEF in DMF compared to THF and dioxane (Figure 3). An increase in PEF can be attributed to an increase in pyrene aggregation or a decrease in solvent viscosity. To investigate the effect of the latter, the fluorescence spectra of Py-PDMS in THF (*η* = 0.47 mPa.s) were compared to those acquired in dioxane (*η* = 1.37 mPa.s), a more viscous solvent with a chemical structure similar to that of THF [17]. As shown in Figure 3A,C, the spectra acquired in THF and dioxane are similar, implying similar PEF. Thus, the difference in PEF between the spectra obtained in THF and DMF should not be attributed to a difference in solvent viscosity. Rather, as will be demonstrated below from the analysis of the fluorescence decays, the lower PEF observed in DMF stems from the absence of pyrene aggregates generated by H-bonds in THF and dioxane between the amide linkers connecting the pyrene labels to the polysiloxane backbone. Here again, the analysis of the fluorescence spectra is complicated by the presence of an additional pyrene species, with the pyrene aggregates emitting as excimers upon direct excitation.

**Figure 4:**
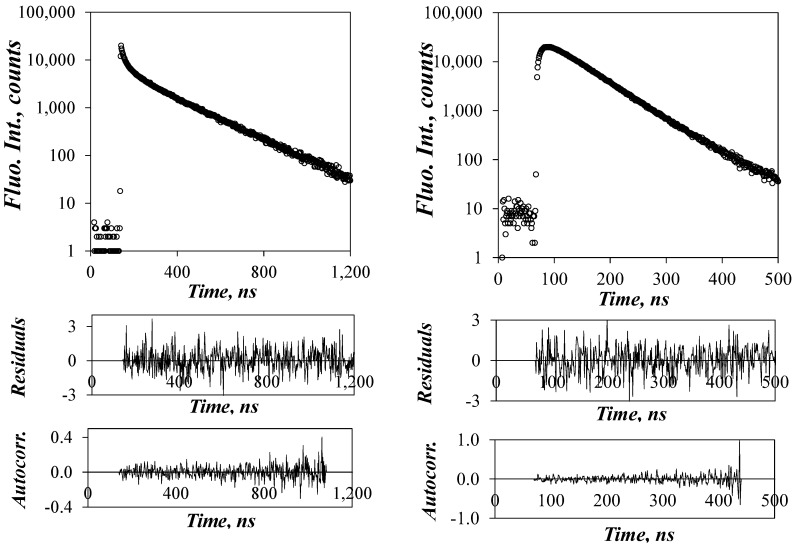
TRF decays of the pyrene monomer (**left**, λ_em_ = 375 nm) and excimer (**right**, λ_em_ = 510 nm) for the Py(5.2)-PDMS sample in THF fitted according to the MFA. λ_ex_ = 344 nm.

The fluorescence decays of the Py-PDMS solutions were acquired and fitted according to the MFA. The MFA equations were derived by noting that any fluorescence decay can always be fitted with a sum of exponentials. This insight was applied to fit the fluorescence decay of the pyrene monomer with the sum of exponentials given in Equation (2), where one of the exponentials had a lifetime (*τ*_M_) matching that of isolated pyrenes (*P*_free_*) and the other exponentials would represent pyrene excimer formation by diffusive encounters. The sum of exponentials describing excimer formation could be processed according to the standard kinetic scheme for excimer formation to yield a mathematical expression for the fluorescence decay of the excimer as shown with Equation (3). Global analysis of the monomer and excimer decays by optimizing both the pre-exponential factors and decay times ensured that all relevant parameters describing the kinetics of pyrene excimer formation could be retrieved with good accuracy. The fits were excellent, yielding *χ*^2^ values that were always <1.30 and plots of the residuals and autocorrelation of the residuals that were randomly distributed around zero. An example of the fluorescence decay fits is shown in Figure 4. All parameters retrieved from the MFA of the fluorescence decays are listed in Tables S1–S3 in Supplementary Materials.

Fitting the fluorescence decays yielded the average rate constant <*k*> for PEF calculated with Equation (4) and the molar fractions of all pyrene species present in solution. These included the pyrene labels that formed excimer by diffusion (*Py*_diff_*), those that were aggregated and formed excimer by direct excitation of a dimer where the pyrene labels were well (*E*0*) or poorly (EL*) aligned, and those that were unable to form excimer (*Py*_free_*) (either because they were unattached and free in solution (PAA*), located in a pyrene-deficient region of the polymer or attached to a short oligomer (Py*-ODMS)). These pyrene species had molar fractions *f*_diff_, *f*_E0_, *f*_EL_, *f*_free_, *f*_PAA_, and *f*_ODMS_, respectively.

**Figure 5:**
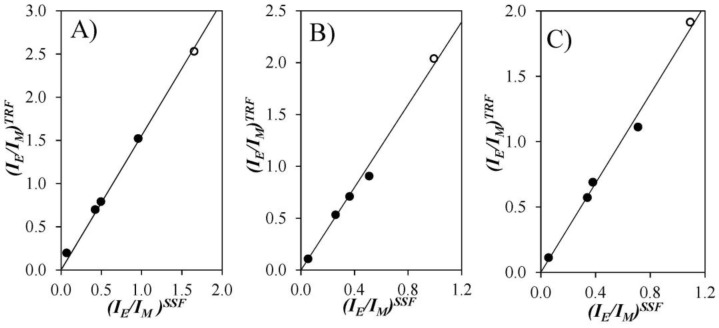
Plot of (*I*_E_/*I*_M_)^TRF^ as a function of (*I*_E_/*I*_M_)^SSF^ in (**A**) THF; (**B**) DMF; and (**C**) dioxane. (●) unfractionated and (○) fractionated Py(3.5)-PDMS samples.

As shown in Equation (6), the MFA parameters can be combined to provide an absolute measure of the ratio of the fluorescence intensity from the excimer and monomer, (*I*_E_/*I*_M_)^TRF^. Figure 5 shows plots of (*I*_E_/*I*_M_)^TRF^ versus (*I*_E_/*I*_M_)^SSF^ (obtained from analyses of all fluorescence spectra) in THF, DMF, and dioxane. The linear relationship suggests that the parameters retrieved from the MFA of the TRF decays describe accurately the fluorescence behavior of the Py-PDMS samples. Furthermore, the point resulting from the ratio (*I*_E_/*I*_M_)^TRF^ and (*I*_E_/*I*_M_)^SSF^ for the fractionated sample, Py(3.5)-PDMS(f), also fell on the line of best fit for the unfractionated samples.

While the MFA parameters in Figure 5 support the attribution of the various pyrenyl species, the presence of Py-ODMS and residual PAA in the solution with pyrene labels that could not form excimer complicated the interpretation of <*k*^MF^>^blob^, which accounts for the change in pyrene content of the different Py-PDMS samples while assuming that all pyrene labels are covalently attached to PDMS. As shown in Equation (5), <*k*^MF^>^blob^ depends on *x*, the molar fraction of structural units in PDMS bearing a pyrene label and *f*_Mfree_, the molar fraction of pyrene labels bound to PDMS that do not form excimer and are probed in the monomer decay. Unfortunately, because the monomeric emissions from the *Py*_free_*, PAA*, and Py*-ODMS species exhibit a long lifetime of about 250 ns in THF, their individual contributions could not be isolated through MFA of the TRF decays; only the sum could be ascertained.

The derivation of *x* was mentioned in the Experimental section and is described in details in the Supporting Information. The derivation of *f*_Mfree_ takes advantage of the fact that the MF and GPC analyses in Table 1 yield the sum of *f*_free_ + *f*_ODMS_ + *f*_PAA_ and of *f*_ODMS_ + *f*_PAA_, respectively. Subtracting the latter from the former yielded *f*_free_ which could then be utilized to determine the molar fractions, *f*_diff_*, *f*_free_*, and *f*_agg_* (= *f*_E0_* + *f*_EL_*), the latter fractions representing all of the pyrene species attached to the polymeric PDMS sample. They correspond to the main component of the GPC traces shown in Figure 2. In turn, the fractions *f*_diff_* and *f*_free_* were employed to calculate *f*_Mfree_ (see Equation (S20)), which was then employed in Equation (5) to determine <*k*^MF^>^blob^.

**Figure 6:**
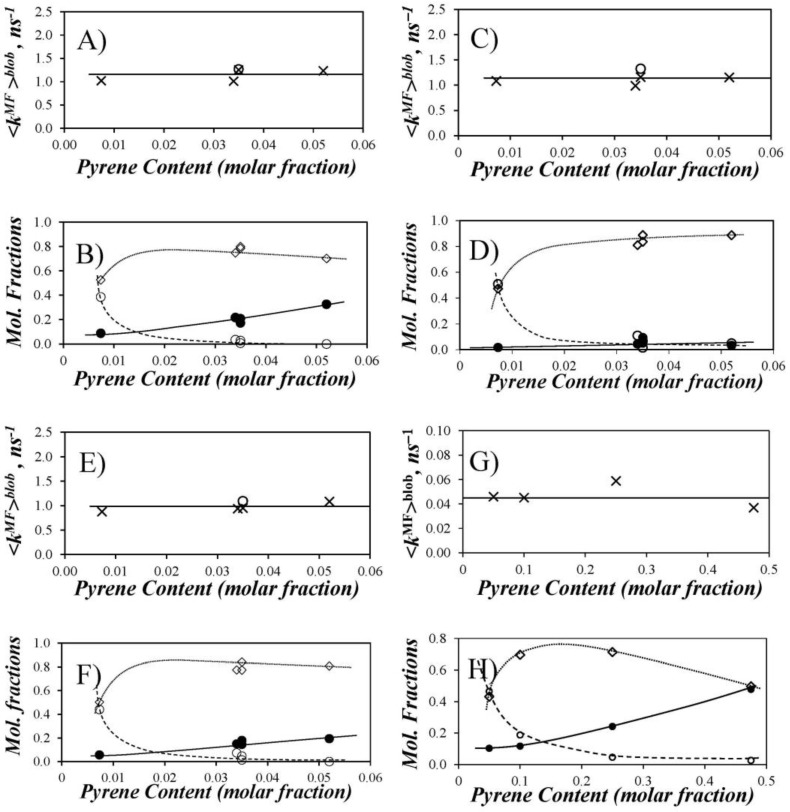
Summary of (**A**, **C**, **E**, and **G**) <*k*^MF^>^blob^ (×, unfractionated Py-PDMS samples; ○, Py(3.5)-PDMS(f)) and (**B**, **D**, **F**, and **H**) the molar fractions (○) *f*_free_*, (◇) *f*_diff_*, and (●) *f*_agg_* as a function of the molar percentage of pyrenyl labels for Py-PDMS in (**A**,**B**) THF; (**C**,**D**) DMF; (**E**,**F**) dioxane and (**G**,**H**) Py-PIMA in THF.

Thus, *f*_diff_*, *f*_free_*, and *f*_agg_* and the average rate constant <*k*^MF^>^blob^ were plotted in Figure 6 as a function of pyrene content for the Py-PDMS samples in THF, DMF, and dioxane. For comparison purposes, similar trends are plotted for the previously reported data in THF with Py-PIMA [11], a polymer whose backbone is expected to be much more rigid than that of PDMS. The profiles of the molar fractions display some common features. At low pyrene content, a large fraction of the lumophore groups (*f*_free_*) did not form excimer, and the polymers showed very little pyrene aggregation (*f*_agg_* ~ 0). As reflected by the increases in *f*_diff_* and *f*_agg_* (except for Py-PDMS in DMF where *f*_agg_* ~ 0), more excimer was produced by diffusive encounters when the pyrene content was increased to intermediate values. At the highest pyrene contents explored, no isolated pyrene remained in solution (i.e., *f*_free_* = 0). As found for the Py-PDMS samples in DMF, *f*_diff_* can pass through a maximum or remain constant depending on the extent of pyrene aggregation. The absence of significant aggregation in DMF suggests that the amide linkers connecting 1-pyreneacetic acid to the 3-aminopropyl side chains of the PDMS substrate induces some H-bonding for Py-PDMS in THF and dioxane. Nevertheless, the level of aggregation remained moderate for the Py-PDMS samples even in these solvents; *f*_agg_ remained <0.35 for all solvents over the range of pyrene contents investigated.

The pyrene content (*x*(*f*_free_ = 0)), where *f*_free_* reaches zero in the plots shown in Figure 6, marks the point where all pyrene labels are able to form an excimer. This point is an indicator of chain flexibility because a more flexible polymer will allow all pyrene labels to form an excimer more readily than a stiffer one. A comparison of the *x*(*f*_free_ = 0) value of 0.03 for the Py-PDMS series in THF, DMF, and dioxane to that of 0.25 (10 times larger) for Py-PIMA in THF [11] clearly underscores the difference in flexibility of the two backbones. PIMA, with its succinic anhydride and succinimide rings being parts of the polymer backbone, is much stiffer than PDMS. Only at very high pyrene contents in Py-PIMA, with *x*(*f*_free_ = 0) = 0.25, were all pyrene labels capable of forming an excimer.

Information about the LRPCD of PDMS was obtained by calculating <*k*^MF^>^blob^ for each Py-PDMS sample. The plots of <*k*^MF^>^blob^ as a function of pyrene content indicate that <*k*^MF^>^blob^ does not depend on pyrene content (Figure 6). The <*k*^MF^>^blob^ values at 22 °C were 1.16 (±0.13) ns^−1^ in THF, 1.14 (±0.12) ns^−1^ in DMF, and 0.99 (±0.10) ns^−1^ in dioxane. At least within the limited range explored, <*k*^MF^>^blob^ does not depend on viscosity either (0.47, 0.79, and 1.37 mPa·s for THF, DMF, and dioxane at 25 °C, respectively) [17].

**Figure 7:**
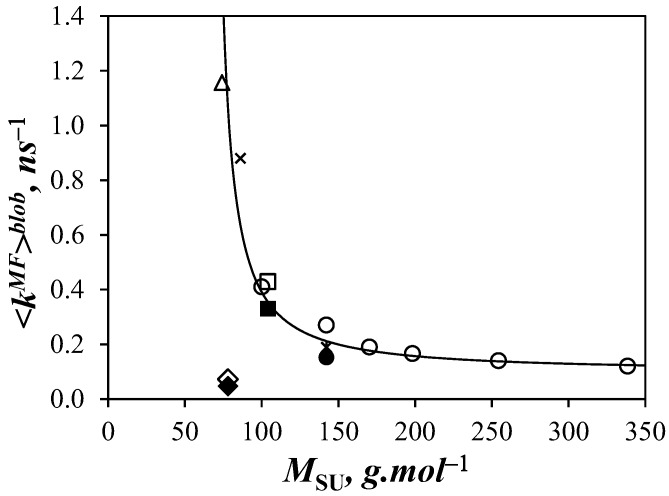
Plot of <*k*^MF^>^blob^ as a function of average structural unit (*M*_SU_) in THF: (△) Py-PDMS; (◆) Py-PIMA; (◇) Py-PIMA scaled by 1.54; (●) PyMeO-X(3)-PC4MA; (◯) PyBut-X(6)-PC*y*MA series with *y* = 1, 4, 6, 8, 12, 18; (■) PyMeN-X(3)-PS; (☐) PyBut-X(6)-PS; (✕) Py-PC1A; (✴) Py-PC4TMA [1,2].

<*k*^MF^>^blob^ for Py-PDMS in THF was then compared to the values found with other polymeric backbones from previous studies [9,11]. The data are plotted in Figure 7 as a function of the molar mass of the structural units (*M*_SU_) of the different polymers. In general, <*k*^MF^>^blob^ was found to decrease quasi-exponentially with increasing *M*_SU_. However, an obvious exception is the comportment of PIMA whose rigid backbone yielded the lowest <*k*^MF^>^blob^ value despite PIMA having one of the smallest *M*_SU._ With its low *M*_SU_, PDMS resulted in the largest <*k*^MF^>^blob^ value obtained reported for any polymer thus far (1.26 ns^−1^), being 2.9 and 27 times larger than for polystyrene (0.43 ns^−1^) and PIMA (0.047 ns^−1^) in THF, respectively. Nevertheless, the overall trend is reasonable in that bulkier and less flexible structural units slow LRPCD.

Another interesting feature of Figure 7 is that, except for PIMA, the polymer backbones yield <*k*^MF^>^blob^ values that seem to cluster around a master curve. If this preliminary trend proves correct, the LRPCD of any polymer in THF should be quantitatively predictable from knowledge of its *M*_SU_ using Figure 7 as a calibration curve.

<*k*^MF^>^blob^ = 21.5 × (*M*_SU_ − 65)^−1.21^ (7)

While it is reasonable and expected that <*k*^MF^>^blob^ for PDMS, a polymer well-known for its flexibility [10,18], is greater than for PS in THF, the 2.9 fold difference is much smaller than that reported for the dynamics of pyrene end-labeled monodisperse PDMS and PS polymers in toluene [19]. Thus, the fact that the LRPCD probed by PDMS constructs that are monodisperse and end-labeled are thrice the value for polydisperse and randomly labeled PDMS with pyrene may be a consequence of the different bond angles of the PDMS backbone: the Si–O–Si bond angle is 146° whereas that of O–Si–O is 108° [18]. This difference implies that a PDMS chain in its most energetically stable *all trans*-conformation would be coiled, whereas the backbone of a PS chain in the same *all trans*-conformation would be fully extended because its C–C–C bond angles are near 109°. Consequently, the ends of a PDMS chain in solution are much closer to each other than those of a PS chain. For experiments in which the dynamics of end-to-end cyclizations are being measured, PDMS allows faster rates of encounter than does PS, and, thus, an apparently larger difference in LRPCD. However, this effect is expected to be much less pronounced for pyrene labels that are randomly distributed along the PDMS chains, and the difference in LRPCD is expected (and found) to be smaller.

## 4. Conclusions

As anticipated from *T*_g_ measurements of PDMS in the solid-state [10,18], we find that a polysiloxane backbone yields fast LRPCD in solution. Furthermore, this study confirms that the Model Free Analysis of TRF decays of polymers randomly labeled with pyrene yields reliable and quantitative information about the relative magnitudes of the dynamic processes experienced by different polymeric chains in solution. The correlation between <*k*^MF^>^blob^ and *M*_SU_ for several polymers suggests that it may be possible to use Figure 7 as an *a priori* tool to calculate <*k*^MF^>^blob^ in a variety of different polymers. It will be interesting in future studies to determine the extent to which this approach is valid. If shown to be widely applicable, this approach would provide an important scale for ranking LRPCD that would be useful in numerous fields, such as for protein folding, where LRPCD controls the early stages of folding for extended polypeptides in coiled conformations [7].

Another interesting finding of this study is that it may have uncovered a problem associated with the use of end-to-end cyclization experiments to characterize LRPCD. In the case of polysiloxanes, where the backbone adopts a more coiled conformation than the polymeric backbone obtained with more traditional vinyl monomers, the end groups may be much closer than expected, thus yielding apparently faster kinetics of end-to-end cyclization that would be interpreted incorrectly as faster LRPCD. Because excimer formation between pyrene labels randomly attached to a polymer is able to probe the dynamics of the entire polymer chain and is localized within a few tens of structural units, it is much less prone to artefacts induced by special backbone conformations that lead to a shorter average end-to-end distance. Comparison of the LRPCD between two polymer backbones by end-to-end cyclization experiments would require that the two polymers have the same contour length and the same end-to-end distance. That is certainly not the case for a polystyrene and a member of the polysiloxane family! Perhaps most importantly, this study confirms that photophysical investigations of polymers randomly labeled with pyrene are a reliable means to probe LRPCD in solution with a variety of solvents.

## Figures and Tables

**Table 1:** Molar fractions of pyrene species present as Py-PDMS (*f*_PDMS_), Py-ODMS (*f*_ODMS_), and PAA (*f*_PAA_).

Py-PDMS Sample	*f*_PDMS_	*f*_ODMS_	*f*_PAA_
Py(0.9)-PDMS	78	8	14
Py(3.4)-PDMS	88	9	2
Py(3.5)-PDMS	87	10	3
Py(5.2)-PDMS	90	9	1

## Supplementary Materials

The supplementary materials are available online at http://www.mdpi.com/2073-4360/10/4/345/s1. Characterization of the Py-PDMS and BPAA samples by ^1^H and ^13^C NMR and positive ion electrospray MS; calculations for the determination of the molar fractions of the different pyrene species covalently attached onto the polymer; parameters retrieved from the MFA of the fluorescence decays.
