# Supplementary material for: Long Range Polymer Chain Dynamics of Highly Flexible Polysiloxane in Solution Probed by Pyrene Excimer Fluorescence
Source: Polymers (Basel). 2018 Mar 21;10(4):345. doi: 10.3390/polym10040345 (PMC6414900; doi:10.3390/polym10040345)
Supplement: Supplementary file 1 [file polymers-10-00345-s001.pdf]

Supporting Information for

Long Range Polymer Chain Dynamics of Highly Flexible Polysiloxane in Solution Probed by Pyrene Excimer Fluorescence

Janine L. Thoma, Jean Duhamel*

Institute for Polymer Research, Waterloo Institute for Nanotechnology, Department of Chemistry, University of Waterloo, Waterloo, ON N2L 3G1, Canada

Michael J. Bertocchi,[b] Richard G. Weiss[b,c]*

[b] Department of Chemistry and [c] Institute for Soft Matter

Synthesis and Metrology, Georgetown University, Washington, D.C. 20057-1227, USA

*To Whom correspondence should be addressed.

TABLE OF CONTENTS

NMR spectra of 1-pyreneacetic acid and Py-PDMS samples ……………………………….. 2 – 3

^{1}H NMR, electrospray ionization mass, and fluorescence spectra and time-resolved fluorescence decay of *N*-butyl 1-pyreneacetamide……………………………………………………. 4 – 5

Derivation for the molar fraction of pyrene labels attached onto PDMS………………….... 6 – 7

Derivation for the molar fraction of pyrene species attached onto PDMS ………………….. 8 – 9

Parameters retrieved from the MFA of the fluorescence decays…………………………. 10 – 13

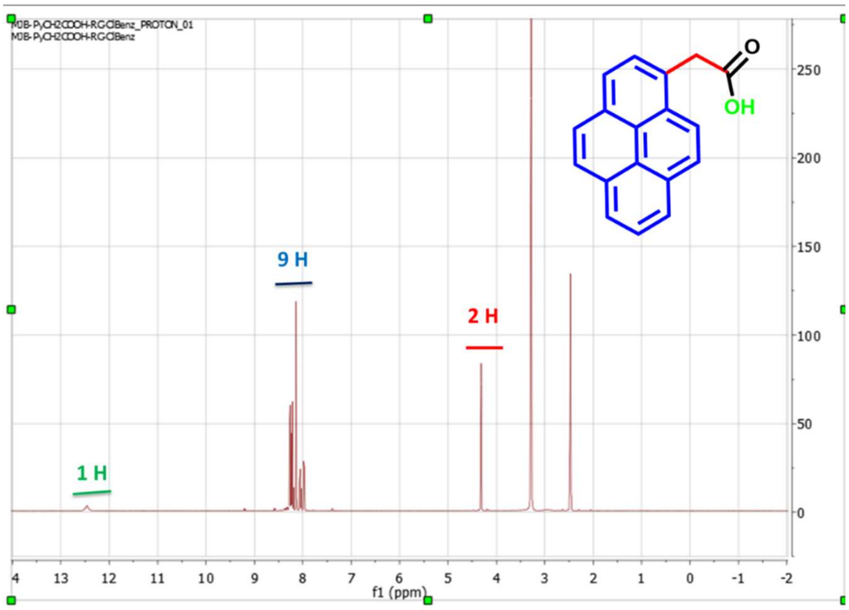

Figure S1. ^{1}H NMR spectrum of (1-pyrene)acetic acid. (DMSO-d_6, 400 MHz), δ 4.21 (s, 2H), 7.8-8.27 (m, 9H), 12.51 (s, 1H).

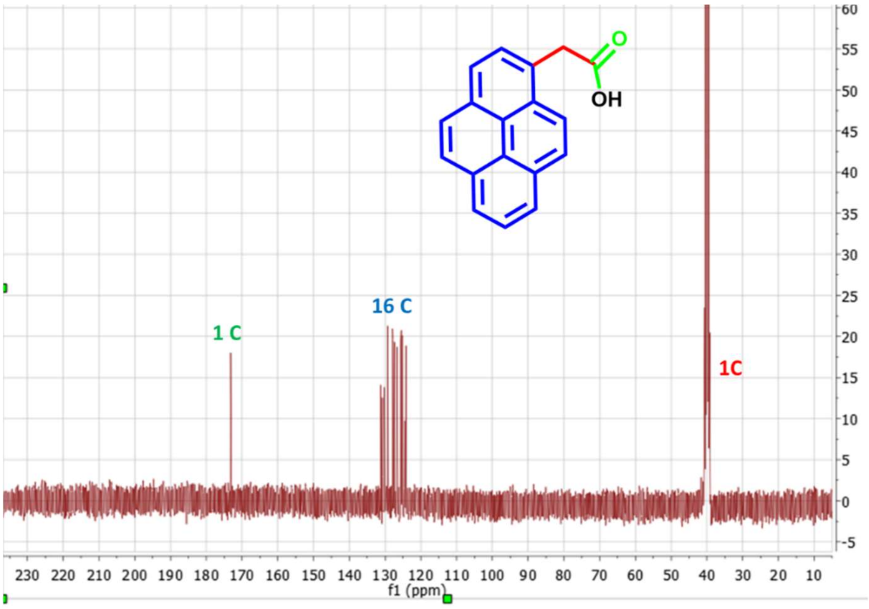

Figure S2. ^{13}C NMR spectrum of (1-pyrene)acetic acid. (DMSO-d_6, 400 MHz), δ 173.1, 131.2, 130.7, 130.3, 130.2, 129.4, 129.2, 127.9, 127.8, 127.4, 126.6, 125.5, 125.5, 125.2, 124.5, 124.3, 124.2, 39.2.

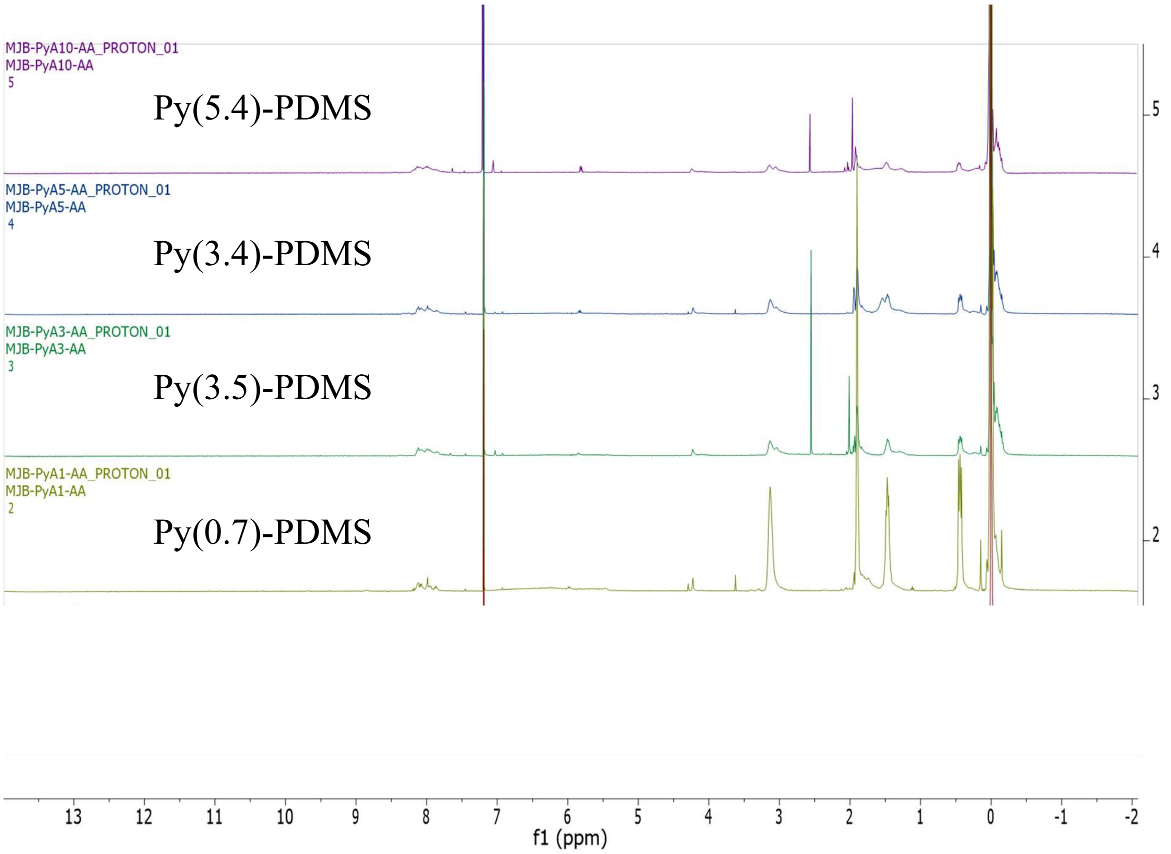

Figure S3. ^{1}H NMR spectra of the Py(X)-PDMS samples in $CDCl_3$

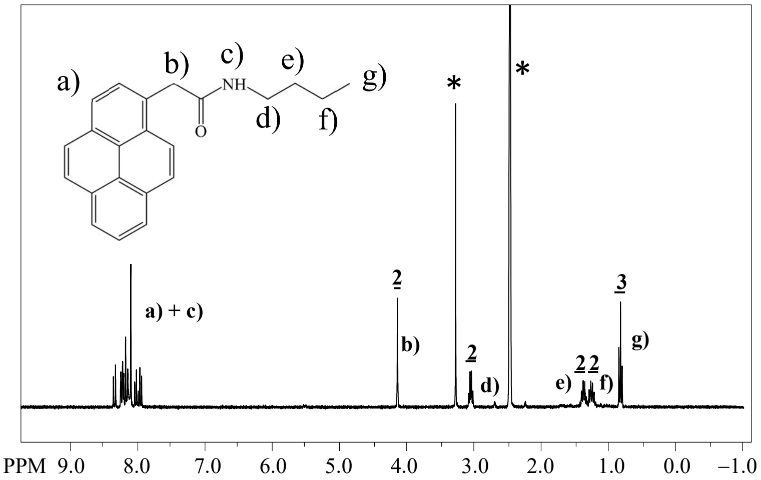

Figure S4. ^{1}H NMR spectra of *N*-butyl-1-pyreneacetamide. (DMSO-d_6 300MHz), δ 0.81 (t, 3H), 1.25 (tq, 2H), 1.36 (tt, 2H), 3.05 (dt, 2H), 4.14 (s, 2H), 8.03 (t, 1H), 7.94-8.39 (m, 9H). Residual solvent peaks at δ 3.27 ppm for H_2O and δ 2.46 ppm for DMSO.

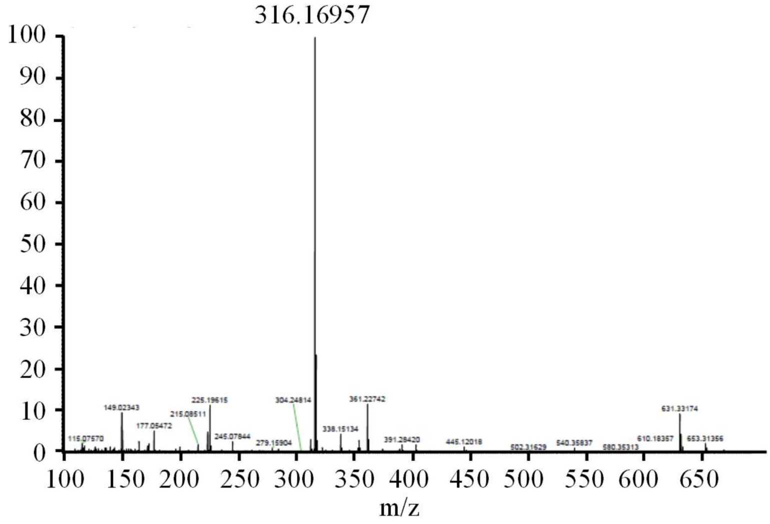

Figure S5. Positive ion Electrospray mass spectrum of *N*-butyl-1-pyreneacetamide in 1:1 methanol/water + 0.1% formic acid. (M_{theo} = 316.16959 g.mol^{-1}, M_{exp} = 316.16825 g.mol^{-1})

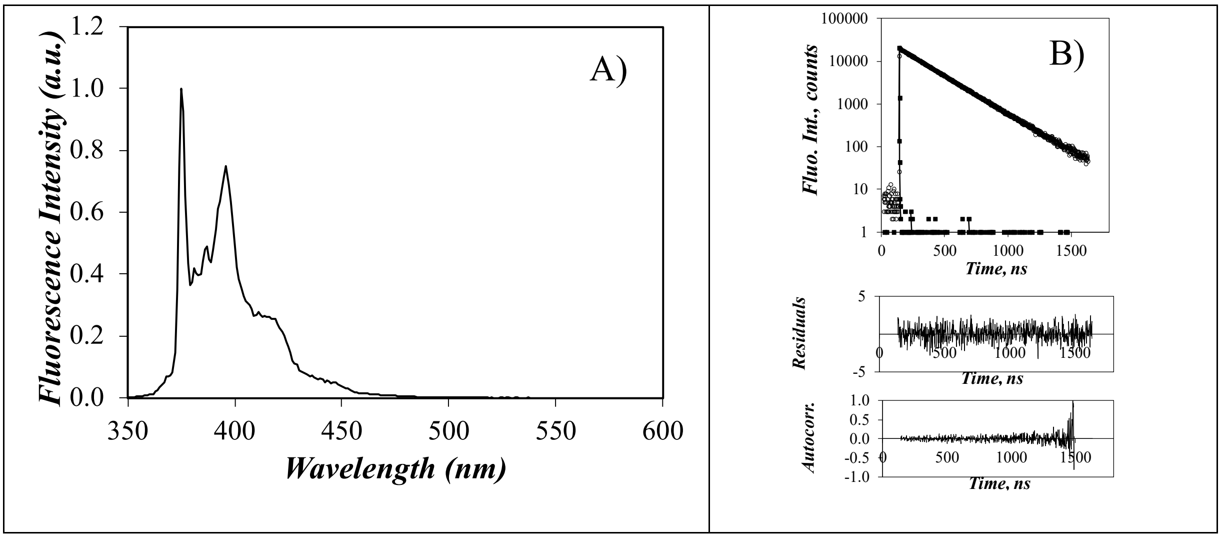

Figure S6. A) Fluorescence spectrum of *N*-butyl-1-pyreneacetamide (BPAA) in THF and B) fluorescence decay of BPAA in THF acquired at λ_{em} = 375 nm and fitted with a single exponential (χ^2 = 0.96 τ_M = 240 ns). [BPAA] = 2.5×10^{-6} M, λ_{ex} = 344 nm.

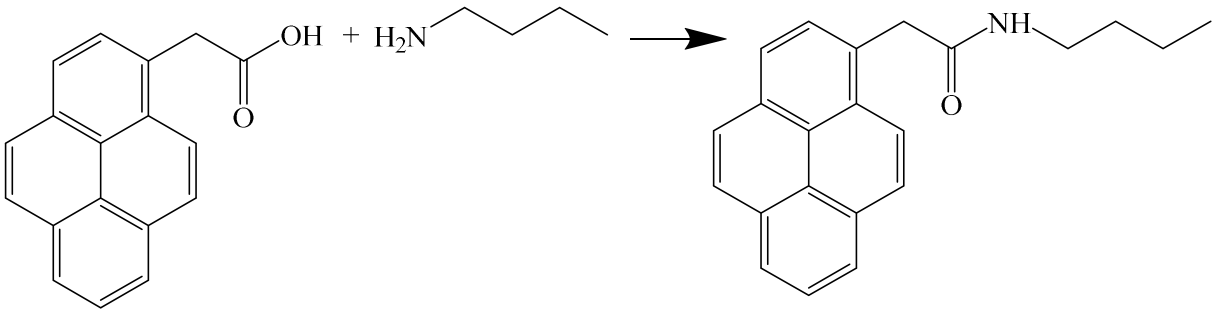

Scheme S1. Synthesis of *N*-butyl-1-pyreneacetamide.

Determination of the molar fraction of the pyrene labels covalently attached onto the polymeric fraction of the PDMS sample

UV-Vis absorption measurements conducted on Py-PDMS solutions of known massic concentration yield the pyrene content λ_{Py} in mol.g^{-1} whose expression is given in Equation S1.

$$\lambda_{Py} = \frac{[Py-PDMS]+[Py-ODMS]+[PAA]}{([Py-PDMS]+[Py-ODMS])M_{SiNPy}+[SiN]M_{SiN}+[Me_2Si]M_{Si}+[PAA]M_{PAA}} \quad \text{(S1)}$$

In Equation S1, ([Py-PDMS] + [Py-ODMS]), [SiN], [Me_2Si], and [PAA] are the molar concentrations of the (3-aminopropyl)methyl siloxane capped with 1-pyrene acetic acid of molar mass M_{SiNPy} (359.48 g.mol^{-1}), the acetyl capped (3-aminopropyl)methyl siloxane of molar mass M_{SiN} (159.26 g.mol^{-1}), the dimethylsiloxane monomers of molar mass M_{Si} (74.15 gmol^{-1}), and 1-pyreneacetic acid of molar mass M_{PAA} (260 g.mol^{-1}), respectively. GPC analysis also provides the molar fractions f_{PAA}, f_{PDMS}, and f_{ODMS} of PAA that is not attached to the polymer and the pyrene labels that are attached onto PDMS and ODMS, respectively. The molar fractions f_{PAA}, f_{PDMS}, and f_{ODMS} determined from GPC analysis can be related to the concentrations [Py-PDMS], [Py-ODMS]), and [PAA] used in Equation S1 according to Equation S2.

$$\frac{[PAA]}{[Py-PDMS]+[Py-ODMS]} = \frac{f_{PAA}}{f_{PDMS}+f_{ODMS}} \quad \text{(S2)}$$

Furthermore, the molar fraction x of pyrene labels covalently attached onto the PDMS substrate is given by Equation S3.

$$x = \frac{[Py-PDMS]+[Py-ODMS]}{[Py-PDMS]+[Py-ODMS]+[SiN]+[Me_2Si]} \quad \text{(S3)}$$

Finally, the molar fraction α of (3-aminopropyl)methyl siloxane units in the PDMS sample was expressed according to Equation S4.

$$\alpha = \frac{[Py-PDMS]+[Py-ODMS]+[SiN]}{[Py-PDMS]+[Py-ODMS]+[SiN]+[Me_2Si]} \tag{S4}$$

Equations S2 – S4 can be re-arranged to determine the concentrations [PAA], [SiN], and [Me_2Si] as a function of the sum of concentrations ([Py-PDMS] + [Py-ODMS]) as shown hereafter.

$$[PAA] = \frac{f_{PAA}}{f_{PDMS}+f_{ODMS}}([Py-PDMS]+[Py-ODMS]) \tag{S5}$$

$$[SiN] = \left(\frac{\alpha}{x}-1\right)([Py-PDMS]+[Py-ODMS]) \tag{S6}$$

$$[Me_2Si] = \frac{1-\alpha}{x}([Py-PDMS]+[Py-ODMS]) \tag{S7}$$

Combining Equations S2-S4 with Equation S1 yields the molar fraction x of pyrene labels covalently attached onto the PDMS samples as shown in Equation S8.

$$x = \frac{\alpha M_{SiN}+(1-\alpha)M_{Me2Si}}{\frac{1}{\lambda_{Py}}+\frac{f_{PAA}}{f_{PDMS}+f_{ODMS}}\left(\frac{1}{\lambda_{Py}}-M_{PAA}\right)-M_{PySiN}+M_{SiN}} \tag{S8}$$

Derivation of the molar fractions of the different pyrene species covalently attached onto the polymeric fraction of the PDMS sample

Ideally, MFA of the fluorescence decays should yield the molar fractions of all the pyrene species present in the solution as shown in Equation S9-14.

$$f_{diff} = \frac{[Py_{diff}]_o}{[Py_{diff}]_o + [Py_{free}]_o + [Py - ODMS]_o + [PAA]_o + [E0]_o + [EL]_o} \quad \text{(S9)}$$

$$f_{diff} = \frac{[Py_{free}]_o}{[Py_{diff}]_o + [Py_{free}]_o + [Py - ODMS]_o + [PAA]_o + [E0]_o + [EL]_o} \quad \text{(S10)}$$

$$f_{ODMS} = \frac{[Py - ODMS]_o}{[Py_{diff}]_o + [Py_{free}]_o + [Py - ODMS]_o + [PAA]_o + [E0]_o + [EL]_o} \quad \text{(S11)}$$

$$f_{PAA} = \frac{[PAA]_o}{[Py_{diff}]_o + [Py_{free}]_o + [Py - ODMS]_o + [PAA]_o + [E0]_o + [EL]_o} \quad \text{(S12)}$$

$$f_{diff} = \frac{[E0]_o}{[Py_{diff}]_o + [Py_{free}]_o + [Py - ODMS]_o + [PAA]_o + [E0]_o + [EL]_o} \quad \text{(S13)}$$

$$f_{diff} = \frac{[EL]_o}{[Py_{diff}]_o + [Py_{free}]_o + [Py - ODMS]_o + [PAA]_o + [E0]_o + [EL]_o} \quad \text{(S14)}$$

Unfortunately, since the species Py_{free}, *Py*-ODMS, and PAA emit as free non-covalently attached 1-pyreneacetic acid monomer, they are indistinguishable and MFA returns only the sum of their molar fractions.

$$f_{free} + f_{ODMS} + f_{PAA} = \frac{[Py_{free}]_o + [Py - ODMS]_o + [PAA]_o}{[Py_{diff}]_o + [Py_{free}]_o + [Py - ODMS]_o + [PAA]_o + [E0]_o + [EL]_o} \quad \text{(S15)}$$

Fortunately GPC analysis yields the sum $f_{\mathrm{ODMS}}+f_{\mathrm{PAA}}$. Consequently, subtracting the sum $f_{\mathrm{ODMS}}+f_{\mathrm{PAA}}$ obtained by GPC analysis from the sum $f_{\mathrm{free}}+f_{\mathrm{ODMS}}+f_{\mathrm{PAA}}$ obtained by MFA of the TRF decays yields f_{free}*. In turn, the fractions f_{diff}, f_{free}, f_{E0}, and f_{EL} can be rearranged according to Equations Sx-y to yield the molar fraction f_{diff}*, f_{free}*, f_{E0}*, and f_{EL}* of the pyrene species actually bound to the polymer.

$$f_{diff}* = \frac{[Py_{diff}]_o}{[Py_{diff}]_o + [Py_{free}]_o + [E0]_o + [EL]_o} = \frac{f_{diff}}{f_{diff} + f_{free} + f_{E0} + f_{EL}} \quad \text{(S16)}$$

$$f_{free}* = \frac{[Py_{free}]_o}{[Py_{diff}]_o + [Py_{free}]_o + [E0]_o + [EL]_o} \frac{f_{free}}{f_{diff} + f_{free} + f_{E0} + f_{EL}} \quad \text{(S17)}$$

$$f_{E0}* = \frac{[E0]_o}{[Py_{diff}]_o + [Py_{free}]_o + [E0]_o + [EL]_o} = \frac{f_{E0}}{f_{diff} + f_{free} + f_{E0} + f_{EL}} \quad \text{(S18)}$$

$$f_{EL}* = \frac{[EL]_o}{[Py_{diff}]_o + [Py_{free}]_o + [E0]_o + [EL]_o} = \frac{f_{EL}}{f_{diff} + f_{free} + f_{E0} + f_{EL}} \quad \text{(S19)}$$

In turn, f_{Mfree} used to determine $<k^{\mathrm{MF}}>^{\mathrm{blob}}$ in Equation 6 was determined according to Equation S20.

$$f_{Mfree} = \frac{f_{free}*}{f_{diff}* + f_{free}*} \quad \text{(S20)}$$

Parameters retrieved form the global MFA analysis of the decays

Monomer decays THF.

Mol%	τ_1 *(ns)*	a_1	τ_2 *(ns)*	a_2	τ_3 *(ns)*	a_3	τ_M *(ns)*	f_{Mfree}	χ^2
0.7	9.8	0.125	43.2	0.198	134.2	0.120	250	0.557	1.11
3.4	6.2	0.416	26.0	0.263	87.5	0.122	250	0.199	1.07
3.5	4.1	0.409	18.3	0.291	73.5	0.135	250	0.166	1.11
3.5-f	5.3	0.562	24.7	0.318	85.4	0.112	250	0.00825	1.14
5.4	4.8	0.582	20.5	0.232	72.9	0.075	250	0.110	1.08

Excimer decays THF.

Mol%	f_{Ediff}^{E0}	f_{Ediff}^{D}	τ_{E0} *(ns)*	τ_D *(ns)*	f_{EE0}	f_{ED}	χ^2
0.7	0.689	0.168	44.3	96.0	0.131	0.0126	1.11
3.4	0.371	0.404	45.5	57.6	0.125	0.100	1.07
3.5	0.303	0.519	44.6	55.6	0.101	0.0770	1.11
3.5-f	0.326	0.464	42.1	57.4	0.124	0.0855	1.14
5.4	0.318	0.365	43.4	56.0	0.154	0.164	1.08

Molar fractions THF.

Mol%	f_{free}	f_{diff}^{E0}	f_{diff}^{D}	f_{diff}	f_{E0}	f_D	f_{agg}
0.7	0.52	0.0807	0.331	0.412	0.0629	0.00607	0.0690
3.4	0.16	0.339	0.311	0.650	0.104	0.0842	0.189
3.5	0.14	0.446	0.260	0.707	0.0869	0.0662	0.153
3.5-f	0.010	0.461	0.324	0.785	0.124	0.0850	0.209
5.4	0.080	0.336	0.293	0.629	0.142	0.1512	0.293

Pyrene species attached to PDMS in THF

Mol%	f_{free}^{*}	f_{diff}^{*}	f_{agg}^{*}	f_{Mfree}
0.7	0.386	0.526	0.0881	0.423
3.4	0.0348	0.748	0.217	0.0444
3.5	0.0279	0.799	0.173	0.0337

3.5-f	0.00653	0.785	0.209	0.00825
5.4	0	0.701	0.326	0

Monomer decays Dioxane.

Mol%	τ_1 *(ns)*	a_1	τ_2 *(ns)*	a_2	τ_3 *(ns)*	a_3	τ_M *(ns)*	f_{Mfree}	χ^2
0.7	11.4	0.0995	41.9	0.185	130.7	0.127	240	0.589	1.11
3.4	6.3	0.309	21.9	0.317	74.0	0.148	240	0.226	1.11
3.5	7.2	0.407	26.2	0.285	84.7	0.121	240	0.187	1.09
3.5-f	6.2	0.462	24.1	0.399	84.9	0.123	240	0.0162	1.21
5.4	4.2	0.380	15.2	0.384	62.8	0.113	240	0.124	1.19

Excimer decays Dioxane

Mol%	f_{Ediff}^{E0}	f_{Ediff}^{D}	τ_{E0} *(ns)*	τ_D *(ns)*	τ_S *(ns)*	f_{EE0}	f_{ED}	f_{ES}^*	χ^2
0.7	0.650	0.224	43.5	88.8	4.0	0.0985	0	0.0285	1.11
3.4	0.608	0.225	45.4	62.9	4.0	0.107	0.0547	0.00549	1.11
3.5	0.324	0.488	40.4	53.9	4.0	0.0215	0.167	0	1.09
3.5-f	0.161	0.692	31.4	53.6	4.0	0.0665	0.0803	0	1.21
5.4	0.315	0.490	36.4	53.1	4.0	0.00491	0.189	0	1.19

Molar fractions Dioxane

Mol%	f_{free}	f_{diff}^{E0}	f_{diff}^{D}	f_{diff}	f_{E0}	f_D	f_{agg}
0.7	0.556	0.288	0.0994	0.388	0.0437	0	0.043
3.4	0.195	0.489	0.181	0.670	0.0863	0.0440	0.130
3.5	0.157	0.273	0.411	0.684	0.0181	0.141	0.159
3.5-f	0.0139	0.159	0.683	0.841	0.0655	0.0791	0.145
5.4	0.102	0.283	0.440	0.723	0.00441	0.170	0.174

Pyrene species attached to PDMS in dioxane

Mol%	f_{free}^*	f_{diff}^*	f_{agg}^*	f_{Mfree}
0.7	0.440	0.503	0.0568	0.467

3.4	0.0741	0.775	0.151	0.0873
3.5	0.0470	0.773	0.180	0.0573
3.5-f	0.0139	0.841	0.145	0.0162
5.4	0	0.806	0.194	0

Monomer decays DMF.

Mol%	τ_1 *(ns)*	a_1	τ_2 *(ns)*	a_2	τ_3 *(ns)*	a_3	τ_M *(ns)*	f_{Mfree}	χ^2
0.7	9.6	0.103	31.0	0.105	80.5	0.166	205	0.626	1.01
3.4	7.0	0.343	23.0	0.292	69.9	0.121	205	0.244	0.99
3.5	6.0	0.320	18.1	0.353	61.1	0.137	205	0.190	1.15
3.5-f	6.2	0.465	21.1	0.401	64.7	0.117	205	0.0166	1.20
5.4	4.3	0.353	14.3	0.403	58.5	0.0879	205	0.156	1.02

Excimer decays DMF

Mol%	f_{Ediff}^{E0}	f_{Ediff}^{D}	τ_{E0} *(ns)*	τ_D *(ns)*	τ_S *(ns)*	f_{EE0}	f_{ED}	f_{ES}^{*}	χ^2
0.7	0.858	0.0547	47.4	134.5	4.0	0.0370	0	0.0507	1.01
3.4	0.763	0.143	44.8	68.0	4.0	0.0789	0.0111	0.00412	0.99
3.5	0.568	0.328	42.6	57.3	4.0	0.0669	0.0372	0	1.15
3.5-f	0.205	0.697	28.1	50.9	4.0	0.0282	0.0699	0	1.20
5.4	0.332	0.601	39.6	51.7	4.0	0.0115	0.0553	0	1.02

Molar fractions DMF

Mol%	f_{free}	f_{diff}^{E0}	f_{diff}^{D}	f_{diff}	f_{E0}	f_D	f_{agg}
0.7	0.605	0.339	0.0216	0.361	0.0146	0	0.0146
3.4	0.226	0.590	0.111	0.701	0.0611	0.00861	0.0697
3.5	0.174	0.470	0.271	0.740	0.0553	0.0307	0.0860
3.5-f	0.0150	0.201	0.687	0.888	0.0278	0.0688	0.0966
5.4	0.147	0.283	0.513	0.796	0.00980	0.0472	0.0570

Pyrene species attached to PDMS in DMF

Mol%	f^*_{free}	f^*_{diff}	f^*_{agg}	f_{Mfree}
0.7	0.508	0.473	0.0172	0.518
3.4	0.110	0.810	0.0441	0.119
3.5	0.0659	0.837	0.0932	0.0730
3.5-f	0.0150	0.888	0.0516	0.0166
5.4	0.0494	0.887	0.0328	0.0528
